# Bridging the gap: sex-specific differences in Huntington’s disease

**DOI:** 10.1186/s13023-025-04184-3

**Published:** 2026-01-10

**Authors:** Greta Hemicker, Katarína Schwarzová, Samuel Labrecque, Clancy Cerejo, Federico Carbone, Marina Peball, Bernadette Wimmer, Philipp Mahlknecht, Florian Krismer, Atbin Djamshidian, Klaus Seppi, Beatrice Heim

**Affiliations:** 1 Department of Neurology, Medical University of Innsbruck, Innsbruck, Austria; 2 District Hospital Kufstein, Department of Neurology, Kufstein, Austria

**Keywords:** Huntington’s disease, Sex-related differences, Functional capacity

## Abstract

**Introduction:**

Huntington’s disease (HD) is a progressive neurodegenerative disorder caused by an expanded cytosine-adenine-guanine (CAG) trinucleotide repeat on chromosome 4. Exploring sex-related differences in disease manifestation may improve understanding and guide more tailored therapeutic and supportive interventions.

**Objective:**

To investigate sex differences in the clinical presentation and functional outcomes in a single-center HD cohort, including employment status and use of supportive therapies.

**Methods:**

We retrospectively analyzed 102 patients with HD who were regularly seen at the Department of Neurology, Medical University of Innsbruck. Exact CAG repeat length was available for 101 participants. Multinomial logistic regression was applied to assess symptom distribution, access to neurorehabilitation, and the impact on employment status.

**Results:**

We included 44 men and 58 women with genetically confirmed Huntington’s Disease. Among participants with available CAG data (*n* = 101), there was no difference in CAG-Repeat length between male and female patients as well as no differences in the onset of motor or non-motor symptoms (all p-values > 0.05). We found that irritability was significantly more prevalent in female patients (*p* = 0.033). Moreover, women were significantly less likely to be employed than men (*p* < 0.001). No sex differences were observed in the utilization of non-pharmacological therapies such as physiotherapy, occupational therapy, speech therapy, or psychotherapy (*p* > 0.05).

**Conclusion:**

Female patients with HD showed higher rates of irritability and lower workforce participation, suggesting clinically relevant sex-related differences. These findings highlight the importance of considering sex as a factor in both clinical management and social support strategies.

## Introduction

Huntington’s disease (HD) is a progressive neurodegenerative disorder caused by an expanded cytosine-adenine-guanine (CAG) trinucleotide repeat on chromosome 4. Individuals with ≥ 35 repeats carry a lifetime risk of developing HD. The disease manifests with motor symptoms such as chorea, as well as psychiatric and cognitive disturbances often preceding motor onset [1, 2]. Diagnosis relies on genetic testing, family history, and motor assessment, which may be quantified using the Unified Huntington’s Disease Rating Scale (UHDRS) to define diagnostic confidence levels [3–5].

Recent research has highlighted that sex may influence the clinical expression and trajectory of HD, although findings have not always been consistent. Some large-scale registry analyses suggest that women may exhibit more pronounced psychiatric symptoms, including irritability and depression, whereas men may present more frequently with aggression or apathy [6–9]. Additionally, several studies report that female patients may show faster decline in functional capacity, and may experience greater limitations in daily activities and independence [7, 10]. However, CAG repeat length and age of onset appear largely comparable between men and women, pointing toward biological, psychosocial, and societal factors beyond the genetic mutation itself as contributors to sex-specific differences [8, 9].

Management requires a multidisciplinary approach. Physiotherapy, occupational therapy, and speech therapy target motor, functional, and communication deficits, aiming to preserve independence and quality of life [11].

Exercise interventions and psychotherapy further improve motor function, balance, and psychosocial well-being [12, 13].

While most prior research has focused on pharmacological treatments, this study investigates sex-specific differences in clinical presentation and in the use of non-pharmacological therapies. Employment status is additionally analyzed as a surrogate of functional capacity, offering a gender-informed perspective on disease progression and daily functioning in HD. Moreover, HD can significantly impact employment and social participation, and emerging evidence suggests that these impacts may differ by sex. Non-motor symptoms such as depression, apathy, and executive dysfunction can already appear in the prodromal phase and may reduce working ability before motor symptoms become clinically evident [11, 14, 15]. Understanding how men and women differ in clinical presentation and in their participation in supportive therapies may therefore help identify opportunities for more personalized disease management and targeted social support strategies.

The aim of this study was to investigate sex-specific differences in symptom presentation, participation in neurorehabilitation, and employment status in a single-center Austrian HD cohort, thereby contributing to a more nuanced understanding of how HD affects men and women differently in daily life functioning.

## Methods

Data used in this work were generously provided by the participants in the Enroll-HD study and made available by CHDI Foundation, Inc. Enroll-HD is a global clinical research platform designed to facilitate clinical research in HD (Enroll-HD – Clinical Research Platform, 2025). Core datasets are obtained annually from all participants in this multi-center, longitudinal observational study. Each site is required to secure and maintain local ethical approval [16].

The study has been ongoing at our center since 2015 The analysis included all HD patients enrolled at the Medical University of Innsbruck, Austria, up to March 15, 2025 inclusively. In total, retrospective data from 102 patients were analyzed. All participants were ≥ 18 years at enrolment. Genetic testing confirming HD was documented for all 102 participants. However, exact CAG repeat length was available for 101 participants; for one participant, the genetic report specifying the repeat number could not be retrieved.

All participants underwent a semi-structured interview to ask for employment status including disease related work hour reduction or retirement and non-pharmacological neuro-rehabilitative therapy sessions. The neurorehabilitative measures include all kinds of options, such as physio-, occupational-, psycho-, speech- and swallowing therapy but also relaxation and acupuncture.

Clinical evaluation was performed annually using the UHDRS to assess motor symptoms, independency and overall functioning [5]. Functional assessment included activities of daily living such as cleaning, laundry, food preparation, and grocery shopping. In addition, participants completed paper-based questionnaires addressing general health and the impact of HD on daily life. Non-motor symptoms were evaluated with the Problem Behaviour Assessment—short version (PBA-s), covering depression, irritability, cognitive impairment, psychosis, perseverative/obsessive behavior, apathy, and violent or aggressive behavior. These symptoms are rated by how severe they are (from “not at all” to “severe”) and how often they occur (from “never” to “daily”) over the past four weeks. If a symptom hasn’t occurred in the last month, or the rating would not reflect the time since the last visit, the participant can give a third rating of severity, again from “not at all” to “severe.” [17]. The age of onset of each motor and non-motor symptom was systematically recorded.

For each participant, the Disease Burden Score was calculated following the method of Penney Jr. et al. (1997) as current age × (CAG − 35.5), and the CAG-Age-Product (CAP)-Score was derived according to Zhang et al. (2011) using the formula: age × (CAG − 33.66) [18, 19].

### Statistics

Data analysis was conducted using SPSS 30. Normal distribution of the data was verified using the Shapiro-Wilk test, with significance set at *p* > 0.05. Demographic characteristics are reported as frequencies or means ± standard deviations. Differences between groups in demographic and clinical variables were evaluated using parametric and non-parametric tests. To account for multiple testing, p-values were adjusted using Bonferroni correction. Where applicable, analyses were corrected for CAG repeat length and age. Analyses requiring CAG repeat length or CAG-derived scores were restricted to participants with available CAG data (*n* = 101). Employment status was additionally corrected for CAP score. Since employment status comprised three categories, multinomial logistic regression was applied.

## Results

**Fig. 1:**
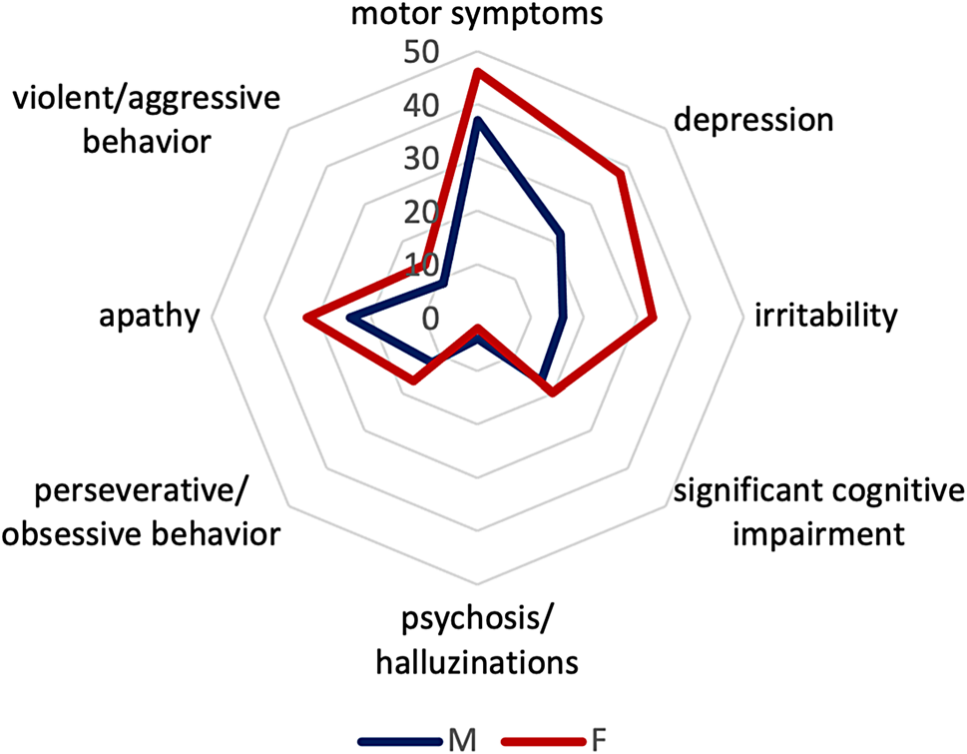
Symptom distribution between sex. Radar chart displaying the symptom distribution between male and female HD patients (*N* = 102)

Demographic data are shown in Table 1. In total, 102 participants with genetically confirmed HD were included. Exact CAG repeat length was available for 101 participants. The mean age was 54.31 (men) and 52,88 (women), respectively. Among participants with available CAG data (*n* = 101), the mean CAG repeat length was 44 in both men and women. No sex-related differences were observed for CAG repeat length, age at onset of motor, or non-motor symptoms. The non-motor symptoms analyzed included depression, irritability, cognitive impairment, psychosis, perseverative/obsessive behavior, apathy, and aggressiveness (see Fig. 1). After correction for CAG repeat length and age, only irritability was more prevalent in women.

**Table 1:** Patient characteristics and cross-sectional analyses comparing male and female HD patients at most recent visit

Characteristics	Men	Women	*p*-value
Patients number (percentage)	44 (43.1%)	58 (56.9%)	
Age at visit	54.31±13.03 (56.0)	52.88±13.94 (55.0)	0.652
CAG repeats*	44.08±4.57 (43)	44.17±3.34 (43)	0.421^a^
Disease burden Score*	436.40±157.12 (442)	432.61±115.06 (429)	0.902
Age of symptom onset	47.37±11.38 (50.0)	45.93±11.43 (45.5)	0.429
Age of motor symptom onset	45.46±12.29 (46.0)	45.98±12.19 (47.0)	0.421
UHDRS-TMS	28.13±22.56 (26.00)	30.37±25.83 (28.00)	0.350^a^
UHDRS-TFC	9.67±4.21 (12.00)	8.85±4.05 (10.00)	0.064^a^
**Clinical symptoms**			
Depression	22 (50%)	38 (65.52%)	0.115^b^
Irritability	16 (36.36%)	33 (56.90%)	**0.033** ^**b**^
Significant cognitive impairment	17 (38.64%)	20 (34.48%)	0.714 ^b^
Motor symptoms	37 (84.09%)	46 (79.31%)	0.539 ^b^
Psychosis/ hallucinations	4 (9.09%)	2 (3.45%)	0.239 ^b^
Perseverative/ obsessive behavior	12 (27.27%)	17 (29.31%)	0.779 ^b^
Apathy	24 (54.55%)	32 (55.17%)	0.873 ^b^
Violent/ aggressive behavior	9 (20.45%)	14 (24.14%)	0.626 ^b^
**Neuro-rehabilitative measures**	15 (34.10%)	26 (44.83%)	0.273 ^b^
Physio	13 (29.55%)	18 (31.03%)	0.871 ^b^
Occupational	3 (6.82%)	9 (15.52%)	0.177 ^b^
Psycho	4 (9.09%)	7 (12.07%)	0.631 ^b^
Speech	7 (15.91%)	11 (18.97%)	0.688 ^b^
Swallowing	1 (2.27%)	3 (5.17%)	0.455 ^b^
Relaxation	9 (20.45%)	1 (1.72%)	0.381 ^b^
Acupuncture	0 (0%)	2 (3.45%)	0.213 ^b^

If not stated differently all values are mean±SD (median)

*available for *n* = 101

^a^Mann-Whitney U test

^b^Pearson chi-square test

SD — standard deviation; CAG — cytosine-adenine-guanine trinucleotide-repeat expansion; UHDRS-TMS — Unified Huntington’s disease Rating Scale-Total Motor Score; UHDRS-TFC — Unified Huntington’s disease Rating Scale-Total functional capacity

Regarding non-pharmacological therapies (physiotherapy, occupational therapy, psychotherapy), no significant sex-specific differences were found.

Employment status differed markedly (see Table 2): men were more often employed full-time, whereas women more frequently worked part-time or were unemployed. These differences remained significant after correction for CAG repeat length or CAP score (CAG-available subgroup, *n* = 101; likelihood ratio test, *p* < 0.001).

**Table 2:** Comparison of the amount of employment between sexes

Amount of employment	Male	Female	Total	*p*-value^a^
Full-time	20 (45.5%)	5 (8.6%)	25 (24.5%)	< 0.001
Part-time	2 (4.5%)	12 (20.7%)	14 (13.7%)	< 0.001
Not employed	20 (45.5%)	40 (69.0%)	60 (58.8%)	< 0.001

p-values refer to Pearson chi-square test *p* < 0.001

^a^p-values refer to multinomial logistic regression corrected for CAG repeats and age

## Discussion

In this cross-sectional analysis of prospectively collected data from an Austrian HD cohort, we examined the influence of sex on clinical presentation, employment status, and participation in neuro-rehabilitative therapies in HD. Male and female patients showed comparable CAG repeat lengths and age at onset. One participant lacked an exact CAG repeat length; thus, CAG-adjusted analyses were performed in *n* = 101. Irritability was more prevalent in women, while other symptoms did not differ significantly, which is consistent with previous studies reporting higher rates of depression or irritability among female patients but no differences in genetic burden or age of onset [6–9].

However, we observed significantly higher rates of irritability in female patients. This finding is consistent with reports indicating that women with HD may experience more pronounced psychiatric symptoms, including emotional dysregulation and mood disturbances, whereas sex differences in motor symptom severity appear less consistent across studies [16–18].

Importantly, some previous work has suggested that women may exhibit a more severe functional phenotype and faster decline in daily independence [7, 10]. This would be in line with our result showing that women with HD had a very low rate of full-time employment (8.6% vs. 45.5% in men). As employment is closely linked to functional capacity and social participation, reduced workforce engagement among women may reflect both clinical burden (e.g., irritability affecting interpersonal functioning) and socioeconomic mechanisms, including gender-based differences in caregiving roles or access to workplace accommodations [9, 14, 20, 21].

Thus, our findings highlight that sex-specific differences in HD are not limited to symptom profiles but extend to broader life impact.

In contrast, we did not observe sex differences in the utilization of non-pharmacological therapies. However, overall rates of physiotherapy, occupational therapy, speech therapy, and psychotherapy were relatively low in our cohort, consistent with previous reports demonstrating underutilization of these interventions despite their potential to maintain functional capacity and improve quality of life (6–8) [21].

Given the absence of disease-modifying treatments, early and continuous access to multidisciplinary rehabilitation should be encouraged for all patients, particularly those at risk for functional decline.

Together, these findings underscore that sex is a relevant factor in the lived experience and functional consequences of HD, with female patients demonstrating more irritability and lower workforce participation. Future studies should investigate whether these differences arise from biological factors, such as hormonal modulation or neurobiological vulnerability, or from gendered social expectations and support structures.

Our results highlight that sex-specific differences in HD primarily manifest in behavioral symptoms and social functioning rather than in genetic burden or motor progression. Recognizing and addressing these differences may support more individualized clinical care and targeted social support strategies.

### Limitations

The main limitation of our study is the relatively small sample size, which may reduce power to detect subtle group differences. Moreover, results may not be generalizable beyond the specific demographic profile of this Austrian cohort.

## Conclusion

Sex-related differences in HD were modest in our cohort, with irritability being more common in women and marked disparities in employment status. These findings emphasize the importance of considering both biological and social factors in patient care.

## Data Availability

The data that support the findings of this study are available from CHDI but restrictions apply to the availability of these data, which were used under license for the current study, and so are not publicly available. Data are however available from the authors upon reasonable request and with permission of CHDI.
